# Spatial Variations in Concentration, Compositions of Glomalin Related Soil Protein in Poplar Plantations in Northeastern China, and Possible Relations with Soil Physicochemical Properties

**DOI:** 10.1155/2014/160403

**Published:** 2014-04-06

**Authors:** Qiong Wang, Yan Wu, Wenjie Wang, Zhaoliang Zhong, Zhongxue Pei, Jie Ren, Huimei Wang, Yuangang Zu

**Affiliations:** ^1^Key Laboratory of Forest Plant Ecology, Northeast Forestry University, Harbin 150040, China; ^2^Daqing Normal University, Daqing 163712, China

## Abstract

Concentration of Glomalin Related Soil Protein is reportedly close related to soil functions, but few data is available for GRSP compositional variations and function related to soil properties. In this paper, soils from 0–20 cm, 20–40 cm, 40–60 cm, 60–80 cm, and 80–100 cm layers were collected in 72 poplar shelterbelts in Songnen Plain (6 regions) for implementing this data shortage. GRSP mainly consists of stretching of O–H, N–H, C–H, C=O, COO–, C–O, and Si–O–Si and bending of C–H and O–H. It has seven fluorescent substances of tyrosine-like protein, tryptophan-like protein, fulvic acid-like, humic acid-like, soluble microbial byproduct-like, nitrobenzoxadiazole-like, and calcofluor white-like, with characteristic X-ray diffraction peak at 2**θ** = 19.8° and 129.3 nm grain size as well as 1.08% low crystallinity. Large spatial variations (intersite and intrasite down profile) were found in either GRSP concentration or these compositional traits. Regression analysis clearly manifested that soil pH should be responsible for these variations. However, negative relations between soil bulk density and GRSP quantity were observed, but not its compositional traits. These basic data in poplar shelterbelt forests are good for understanding the underlying mechanism of GRSP in soil functional maintenance.

## 1. Introduction

Glomalin Related Soil Protein (GRSP) is one kind of glycoprotein which contains metal ions (Fe^3+^) from arbuscular mycorrhizal fungi (AMF) [1, 2]. After its first discovery in 1996, immune fluorescent reactions, chemical stability, extracting methods [1, 2], and wide distribution in variable soil ecosystems and land uses [3–5] are reported. Both abiotic and biological factors such as elevated CO_2_ [6], global warming [7], climate conditions, vegetation types [3, 8], and a variety of agricultural measures could affect GRSP concentration in soils. Technological advances in infrared spectroscopy [9], ultraviolet absorbance spectrum [10], 3D fluorescence spectroscopy [11], X-ray diffraction [12] made it possible to characterize compositional differences in a more detailed way and these techniques favor the recent advances in GRSP composition functionality [9] and complexity [12]. Although relations between GRSP concentration and soil aggregates stability, concentration of soil organic C, N are well known [13, 14], relations between these compositional differences and soil properties are not defined yet, and quantifying GRSP compositional differences can facilitate the definition of their functions in regulating soil physical chemical properties [9, 12].

Worldwide distribution of poplar plantations shows their importance in afforestation practices, and over 710 million hm^2^ of them distribute in China (1/5 of world total), ranking top one in the world [15]. In most cases, poplar shelterbelt forests playing important roles in breaking wind, fixing sands, reducing sound pollution, and capturing carbon in China. As farmland shelterbelts, poplar plantations' function is to defend natural disasters and protect food products in Northeastern China. Symbiotic relationships between over 90% plants and AMF can improve the viability of adversity plants [16]. After AMF infected plants, external hyphae form a huge network in soils [17], transfer information between same or different plants [18], and secret GRSP with function of soil structure modification [19]. There were many studies on farmland shelterbelts ecological protecting function in NE China (e.g., [15]) and intersites and intrasites spatial variations both in GRSP concentration and compositional traits should be a basis for understanding function of GRSP in maintaining optimal soil properties.

GRSP is the typical compounds secreted by AM hyphae [18]; we postulate that large spatial (intrasite down vertical profile and intersite) variations in GRSP (easily extractable GRSP, EE-GRSP; total GRSP, T-GRSP) should exist in both compositional traits and quantity, and some abiotic factors, such as soil pH and so forth, should be responsible for such spatial variations. For approaching this hypothesis, Songnen Plain with a flat topography, relative consistent climate, and abundant of poplar farmland shelterbelts [15] was selected for conducting this study, and 360 soil samples from 72 poplar shelterbelts were sampled for this paper. The aim was to explore the quantity and compositional variations of GRSP between and within sites and down profile, and regression analysis was thereafter adopted for finding their possible contribution to soil physicochemical changes. The following scientific questions will be answered, that is, what are the compositional features of GRSP from the viewpoints of infrared spectrum, fluorescent spectrum, and X-ray diffraction? How large variations in vertical soil profile and different sites were found in these compositional parameters and concentration of GRSP? Which soil parameters were possibly related to such quantity and compositional variations in GRSP?

## 2. Material and Methods

### 2.1. Natural Condition of Study Sites and Preparation of Soil Samples

Songnen Plain is located in the middle of Northeast China and crosses Heilongjiang Province, Jilin Province, and Inner Mongolia Autonomous Region, and the total area is 182,800 km^2^, involving a population of 36 million. Songnen Plain is one of the most important bases for large-scale commodity grain and animal husbandry for China. Poplar shelterbelts forests were planted widely since the launch of national “three-north” shelterbelts project in 1978 [15]. In this paper, poplar shelterbelts were general 3–8 rows trees with mature-aged poplars, and some younger shelterbelts were tens of rows trees. The average of tree density, tree height, and diameter at breast height was, respectively, 3600 trees ha^−1^, 14.91 m, and 23.18 cm.

Soil samples were collected from 72 shelterbelt plantations in 6 typical regions distributed in Songnen Plain (Table 1). Soil classification was based on [20]. In each of the 6 regions, 12 soil profiles were digging out in 12 different shelterbelt forests. In each soil profile, 5 soil samples were collected with a 100 cm^3^ cutting ring from soil layers of 0–20 cm, 20–40 cm, 40–60 cm, 60–80 cm, and 80–100 cm. In total, 360 soil samples (6 × 12 × 5 = 360) were collected. All samples were collected from June to August 2012. After fully air-drying and excluding small stones, distinguishable plant roots, and other debris, samples passed through 0.25 mm sieve were used for laboratory analysis. The reason for this sieve size selection is that larger proportions of GRSP were distributed in 0.21 mm–0.50 mm macroaggregates [21]; moreover, soil samples from 0.25 mm to 1 mm sieves had similar GRSP concentration in China [22].

### 2.2. Determination of Soil Physicochemical Properties

Soil pH was measured in solution with 1 g soil sample in 5 mL deionized water and it was determined with a precise pH meter of Sartorius PB10 (Sartorius, Germany). Soil electrical conductivity (EC) was determined with an EC meter (DDS-307, Shanghai Precision Scientific Instruments Co., Ltd., China). Soil moisture was calculated as (fresh weight − dry weight)/dry weight × 100%. Soil bulk density was calculated as the ratio between air-dried soil mass and the soil volume (400 cm^3^).

### 2.3. Extraction and Determination of GRSP

Extraction and determination of GRSP in soil samples was according to the method described by Wright with a slight improvement [5]. For EE-GRSP, samples of 0.5000 g soil were subjected to extraction with 4 mL of 20 mM citrate, pH = 7.0, and autoclaving for 30 min at 121°C. The T-GRSP was extracted from 0.1000 g of soil with 4 mL of 50 mM citrate, pH = 8.0, and autoclaving for 1 h at 121°C. In both cases, the supernatant was separated by centrifugation at 4000 rpm for 6 min and supernatant was collected. For T-GRSP, the procedure described was repeated several times (autoclaving for 30 min at 121°C) on the same sample until the reddish brown color typical of GRSP disappeared from the supernatant, combining all extracts from a soil sample. The protein content in the crude extract was determined by Bradford assay with bovine serum albumin as the standard. GRSP storage (EE and T) was calculated as the product of GRSP concentration (EE and T), soil depth (20 cm), and soil bulk density.

### 2.4. Determination of Composition Traits of Purified T-GRSP

Soil samples from surface 20 cm soil in each of the 6 regions were mixed as a composite sample for purifications of T-GRSP. Purification of T-GRSP was according to [12]: 1.00 g soil sample was put in 50 mL centrifuge tubes, 8 mL of 50 mM citrate extraction solvent at pH = 8.0. The sample was oscillated for half a minute on the oscillator to ensure mixing thoroughly and extracted at 121°C for 60 min. Then the sample was centrifuged at 4000 rpm for 15 min, and the supernatant was removed to centrifuge cups. The extraction process was repeated, continuous extraction was carried out until the supernatant no longer showed the typical red brown. All the collected extraction was then precipitated by titrating hydrochloric acid and then centrifuged at 4000 rpm for 15 min. The pellets under the centrifuge tube were resolubilized in 0.1 M sodium hydroxide and dialyzed against deionized water for 60 h (dialysis bag, DW = 8000–14000 Da, Scientific Research Special, USA). After dialysis, The purified dialyzate was centrifuged at 10000 rpm for 10 min to remove any extraneous particles. The supernatant was then immediately freeze-dried with vacuum freeze drier (Scientz-10N, Ningbo Scientz Biotechnology Co., Ltd., China).

**Figure 1:**
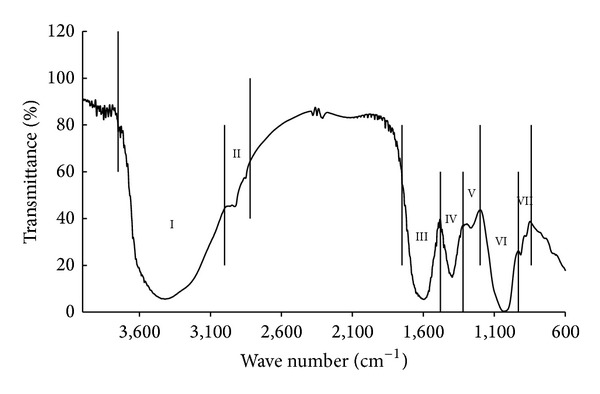
Schematic diagram with partition method of functional traits of GRSP by using infrared spectrum. Note: functional trait I (wave number of 3750–3000 cm^−1^) is a mixture of O–H stretching of carboxylic acid, phenols, alcohols, clay minerals and oxides, N–H stretching of organic amines, amides, and aromatic C–H stretching. Functional trait II (wave number of 3000–2820 cm^−1^) is aliphatic C–H stretching. Functional trait III (wave number of 1750–1480 cm^−1^) is a mixture of C=O stretching of carboxylic acid, ketones and amides, and asymmetric COO– stretching of carboxylic acid salts. Functional trait IV (wave number of 1480–1320 cm^−1^) is a mixture of symmetric COO– stretching of carboxylic acid salts and C–H bending of –CH_2_– and –CH_3_ groups. Functional trait V (wave number of 1320–1200 cm^−1^) is a mixture of C–O stretching and O–H bending of –COOH. Functional trait VI (wave number of 1200–930 cm^−1^) is a mixture of C–O stretching of polysaccharide and Si–O–Si stretching in clay minerals and oxides. Functional trait VII (wave number of 930–840 cm^−1^) is O–H bending of structural OH in clay minerals and oxides.

*Infrared Spectroscopy Measurement*. The samples were diluted with 1% KBr mixing powder and separately pressed to obtain self-supporting disks. Functional traits were determined with IRAffinity-1 infrared spectrometer model (SHIMADZU, Japan) with a spectral range of 4000–500 cm^−1^. For each peak in the spectrum, the absorption peak area could semiquantitatively reflect the concentration of the functional trait matching with this peak. The match between functional traits and peak wave numbers was from [23] and is described as in Figure 1.

*X-Ray Diffraction (XRD) Measurement*. XRD patterns were collected in transmission by using an X-ray diffraction meter (D/Max 2200, Rigaku, Japan) with a rotating anode (Philips) and Cu K*α*1 radiation generated at 30 mA and 40 kV. The range of 2*θ* diffraction angles examined was 10°–40° with steps of 0.02° and a measuring time of 0.3 s per step. In the analysis of XRD data, the original data were rectified using the Jade program to eliminate K*α* and then obtain the XRD pattern for a sample. The upper area (*ac*), which was separated with the smooth curve connecting each point of minimum intensity, corresponded to the crystalline portion, and the lower area was the background containing the amorphous portion (*ab*). The Jade 5 program was used to calculate grain size and relative crystallinity (relative crystallinity = *c*/(*ac* + *ab*)) [24].

*UV Spectrophotometer Measurement*. 1.000 mg freeze-dried T-GRSP samples were put in 10 mL centrifuge tubes, with 1 mL of 0.1 M sodium hydroxide solution dissolved, and diluted 10-fold. It was determined with the UV-visible spectrophotometer of UV-2550 (SHIMADZU Co., Kyoto, Japan), scanning wavelength range: 250–450 nm. OD value and the range of maximum absorption wavelength of T-GRSP were obtained in the figure of the program.

**Figure 2:**
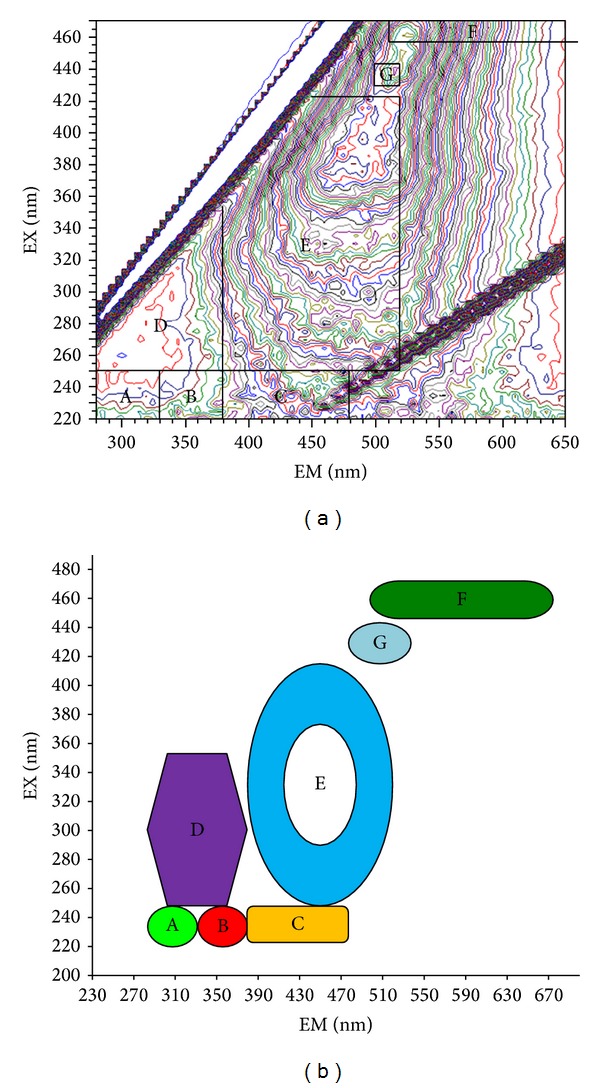
Schematic diagrams with partition method of fluorescent substances of GRSP by using 3D fluorescence spectrum. (a) Fingerprint of the partition of fluorescent substances; (b) pattern of the partition of fluorescent substances. Note: A: tyrosine-like protein, 220–250 nm in excitation and 280–330 nm in emission; B: tryptophan-like protein, 220–250 nm in excitation and 330–380 nm in emission; C: fulvic acid-like, 220–250 nm in excitation and 380–480 nm in emission; D: soluble microbial byproduct-like, 250–360 nm in excitation and 280–380 nm in emission; E: humic acid-like, 250–420 nm in excitation and 380–520 nm in emission; F: nitrobenzoxadiazole-like, 460–470 nm in excitation and 510–650 nm in emission; G: calcofluor white-like, 440 nm in excitation and 500–520 nm in emission.

*Fluorescence Spectrometer Measurement*. 1.000 mg freeze-dried T-GRSP samples were put in 10 mL centrifuge tubes, with 1 mL of 0.1 M sodium hydroxide solution dissolved, and diluted 5-fold, using a Hitachi F-7000 fluorescence spectrometer (Hitachi High Technologies, Tokyo, Japan) with a 700-voltage xenon lamp at room temperature (20 ± 2°C). Readings were collected in ratio mode (S/R) (the default mode of F-7000 fluorescence spectrometer), using a scanning speed of 2400 nm·min^−1^. The scanning ranges were 220–470 nm for excitation and 280–650 nm for emission. The bandpass widths were 5 nm for both excitation and emission. The range of different excitation/emission wavelength of the fluorescence spectra classified the dissolved organic matter in T-GRSP into seven fluorescent materials in accordance with [25] and identification support from Microspheres Online (http://www.microspheres.us/microsphere-basics/fluorochromes-excitation-emission-wavelengths/248.html) (Figure 2).

### 2.5. Data Analysis

Two-way analysis of variance (ANOVA) was used to identify the site- and soil depth-related variations on concentration of T-GRSP and EE-GRSP, soil physicochemical properties, and their possible interactions, with LSD pairwise comparison for multiple comparison. Regression analysis was used to find linear relations between GRSP quantity, compositional traits, and variable soil physicochemical properties. All analysis was performed by SPSS 17.0 (SPSS, USA).

## 3. Results

### 3.1. Variations in Soil Physicochemical Properties: Sites, Soil Layers, and Interaction

Different soil pH, soil bulk density, and soil moisture differed among sites (*P* < 0.01) and among soil layers (*P* < 0.01), while different EC was found only in different soil layers (*P* < 0.01). These site variations were similar at different soil layers because no interaction between sites and soil layers was found (*P* > 0.05) (Table 2).

Estimated marginal means showed the magnitude of site- and soil layer-related variations (Table 2). For different sites, soil pH in Zhaozhou was 1.15-fold higher than the lowest value in Mingshui; soil bulk density in Dumeng was the highest; soil moisture showed a pattern of Fuyu > Mingshui > Zhaodong > Zhaozhou = Lanling > Dumeng; EC in Fuyu was 1.88-fold higher than the lowest site. As deepening of soil, increasing soil pH and soil bulk density but decreasing soil moisture and EC were observed (Table 2).

### 3.2. Variations of GRSP (T-GRSP, EE-GRSP) Concentration in Different Sites and Soil Layers

GRSP (T-GRSP, EE-GRSP) concentration significantly differed at different sites and soil layers. Moreover, as shown for the larger *F* value, site-related variations for all 4 parameters were much larger than those from soil layers (Table 3). The significant interaction between site and soil layer on GRSP showed site variations were different at different soil layers (*P* < 0.01) (Table 3).

The size of site- and soil layer-related variations were observed in estimated marginal means (Table 3). For site variations, T-GRSP showed a similar pattern of Mingshui > Zhaodong > Lanling > Dumeng > Fuyu > Zhaozhou; some variations (e.g., Mingshui and Zhaodong) were statistically significant (*P* < 0.05). For vertical variations, with the deepening of soil layers, decreasing T-GRSP was observed.

For site variations of EE-GRSP both in concentration and storage, the similar pattern of Mingshui > Dumeng > Fuyu > Zhaozhou > Lanling > Zhaodong was found. Some variations (e.g., Mingshui and Dumeng) were statistically significant (*P* < 0.05), while some others (e.g., Dumeng and Fuyu) were not significant (*P* > 0.05). For vertical variations, decreasing EE-GRSP concentration and storage were observed with the deepening of soil layers. EE-GRSP concentration showed that 0–20 cm soil layer was 2.58-fold higher than the lowest value in 80–100 cm soil layer (0.26 mg·g^−1^) (*P* < 0.05).

On average of pooled data, T-GRSP concentration, EE-GRSP concentration, T-GRSP storage, and EE-GRSP storage were, respectively, 3.93 mg·g^−1^, 0.43 mg·g^−1^, 107.89 mg·cm^−2^, and 11.89 mg·cm^−2^ (Table 3).

### 3.3. Site Variations in Composition-Related Functional Traits of Purified T-GRSP

Result of infrared spectroscopy was summarized as follows: for different functional traits, the size of the site variations was different. For example, in the case of functional trait VII (O–H bending), peak value in Zhaozhou was 2.10-fold higher than the lowest value in Fuyu (17.3), while in the case of functional trait III (a mixture of C=O stretching of carboxylic acid, ketones, and amides and asymmetric COO– stretching of carboxylic acid salts), peak value in Zhaodong was only 1.12-fold higher than the lowest in Dumeng (1949.7). Average of functional traits showed that I (8050.9) > III (2039.9) > VI (1277.3) > IV (500.9) > II (258.8) > V (95.1) > VII (25.7) (Table 4).

Result of X-ray diffraction was summarized as follows: diffraction peak position of GRSP was in 2*θ* = 19.8°; the relative crystallization and grain size of T-GRSP were derived from XDR data (Table 5). Dumeng showed the highest grain size (174 nm), followed by Fuyu (138 nm), whereas the lowest value was found in Zhaodong (98 nm). Relative crystallinity showed Mingshui (1.98%) > Lanling (1.35%) > Fuyu (1.05%) > Dumeng (0.73%) > Zhaodong (0.7%) > Zhaozhou (0.69%), and site variations were 2.71-fold. The mean relative crystallinity and grain size were, respectively, 1.08% and 129.3 nm.

Result of OD values and fluorescent intensity at 295 nm was summarized as follows: the wavelength of maximum absorption (OD) was at 294.4 ± 1.8 nm (Table 5), and 1.34-fold variations were observed between Mingshui, the highest site, and Zhaodong, the lowest site (1.02). At 295 nm, the peak excitation fluorescence had 1.49-fold variations between Fuyu and Zhaodong (20.53) (Table 5).

Result of 3D fluorescent spectroscopy was summarized as follows: Table 5 showed that GRSP is a mixture of at least seven fluorescent compounds (tyrosine-like protein, tryptophan-like protein, fulvic acid-like, soluble microbial byproduct-like, humic acid-like, nitrobenzoxadiazole-like, calcofluor white-like), but 1.18–4.50-fold site variations were found. For example, the highest value of tyrosine-like protein was in Zhaozhou (7.78), about 4.50-fold higher than the lowest value in Zhaodong. Humic acid-like had 1.64-fold variations. The highest value of calcofluor white-like was in Dumeng (59.17), and the lowest value was in Zhaodong (50.07) (not detectable in Mingshui and Zhaozhou). Average fluorescent intensity showed a pattern of nitrobenzoxadiazole-like > calcofluor white-like > humic acid-like > fulvic acid-like > tryptophan-like protein > tyrosine-like protein > soluble microbial byproduct-like.

### 3.4. Regression Analysis between GRSP Concentration, Compositional Traits, and Soil Physicochemical Properties

**Figure 3:**
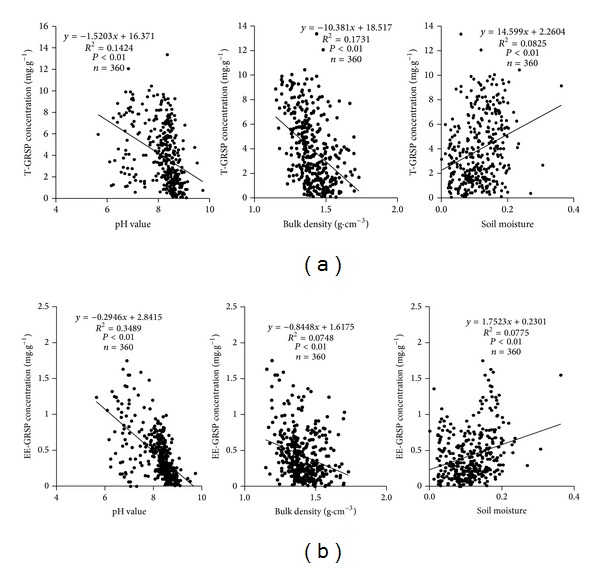
Correlations between soil pH, bulk density, moisture, and T-GRSP concentration (a) and between these parameters and EE-GRSP concentration (b).

Soil pH, soil bulk density, and soil moisture were significantly linearly correlated with T-GRSP and EE-GRSP concentration, respectively (*P* < 0.01). Both T-GRSP and EE-GRSP storage showed a similar pattern (*P* < 0.01) and data were not shown here. *R* ^2^ for linear correlations between pH and quantity of GRSP (T-GRSP, EE-GRSP) ranged from 0.1424 to 0.3489, which was larger than those relations with soil bulk density (*R* ^2^ = 0.1731 to 0.0748) and soil moisture (*R* ^2^ = 0.0825 to 0.0775), showing that pH was the main abiotic factor regulating GRSP concentration changes (Figure 3).

**Figure 4:**
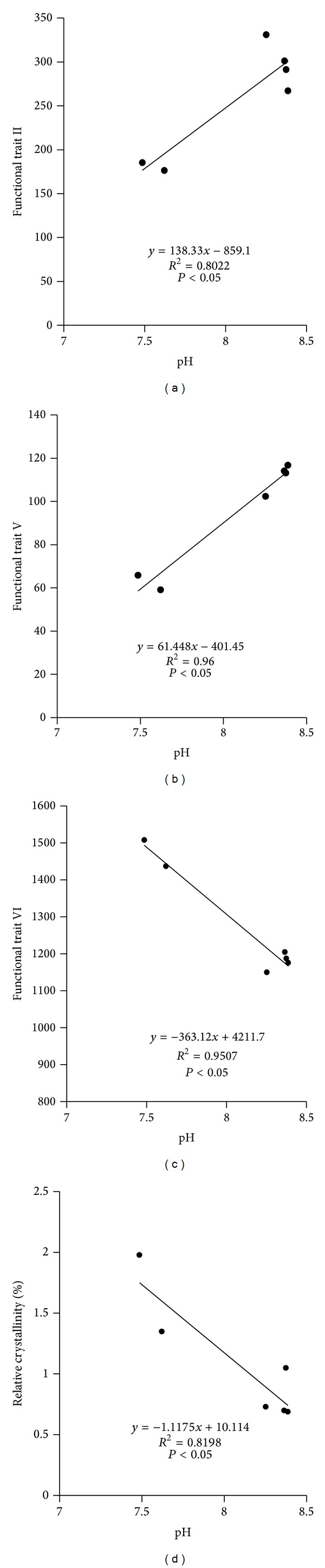
Correlations between soil pH and the concentration of functional trait II, V, VI, and relative crystallinity. Note: functional traits of II, V, and VI are the same as those in Figure 1.

Soil pH was the most important factor affecting compositional traits of T-GRSP, (Figure 4). Soil pH was significantly correlated with infrared functional trait II (aliphatic C–H stretching, positive correlation), trait V (C–O stretching and O–H bending, positive correlation), trait VI (stretching of C–O and Si–O–Si, negative correlation), and relative crystallinity (negative correlation) (*P* < 0.05). However, no correlations were found between soil moisture, soil bulk density, and all compositional traits.

**Figure 5:**
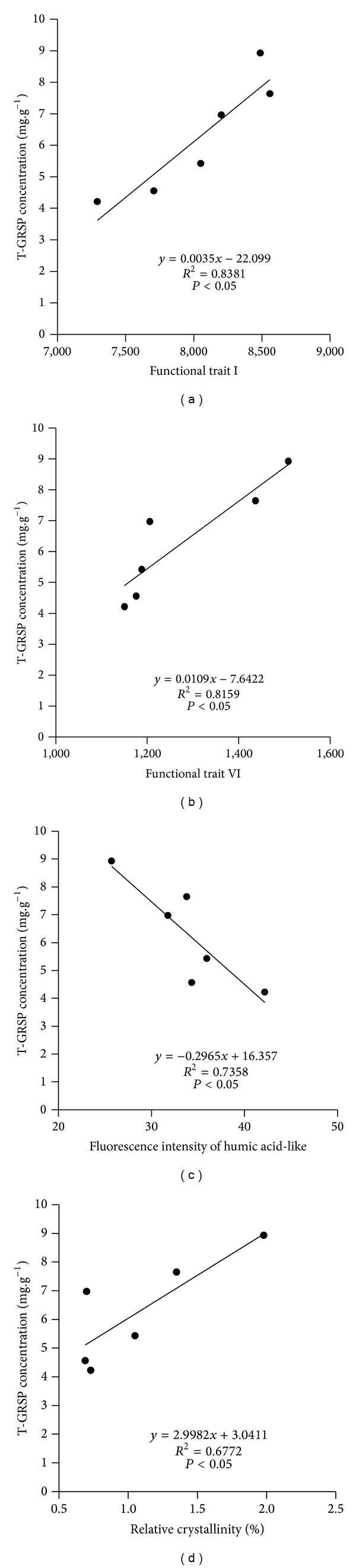
Correlations between T-GRSP concentration and the relative content of functional trait I, VI, fluorescence intensity of humic acid-like, and relative crystallinity. Note: functional traits of I and VI are the same as those in Figure 1.

Significant correlations between composition-related parameters and T-GRSP concentration in soil were also found (Figure 5). Infrared related functional traits including I (stretching of O–H, N–H, C–H), VI (stretching of C–O and Si–O–Si), and X-ray-related functional traits (relative crystallinity) showed significant positive correlations with T-GRSP concentration (*P* < 0.05). 3D fluorescent compounds (humic acid-like) showed the significant negative correlations with GRSP (T-GRSP, EE-GRSP) (*P* < 0.05). T-GRSP storage usually showed a similar pattern (data not shown here).

## 4. Discussion

Since its discovery, GRSP has received wide investigation due to its significant role in the improvement of soil properties [26–28]. Many studies have reported that a variety of environmental factors, such as climate conditions, vegetation types, soil characteristics, atmospheric CO_2_, and land uses, could affect the accumulation of T-GRSP and EE-GRSP in soils [3, 29]. Compared with concentration changes, few studies have focused on composition changes, although their importance gets more and more concerns [9, 12]. The working hypothesis of this paper was testified by the large GRSP spatial variations in both concentration and composition at different sites and vertical profiles are mainly related to soil pH changes, while the concentration of GRSP (instead of its compositional features) was mainly related to soil physics (soil bulk density).

### 4.1. Large Variations in GRSP Concentration in Poplar Shelterbelts: Comparison with References

The marked spatial variations in GRSP concentration were found in different sites and vertical soil profiles, and, as a finding of this paper, we quantified the range of the variations in Songnen Plain in shelterbelt plantations with the same poplar species. In the case of T-GRSP, site variations were 3.04–3.14-fold, and vertical variations were 2.85–2.98 folds. In the case of EE-GRSP, the corresponding variations were, respectively, 3.81–3.95-fold and 2.46–2.58-fold (Table 3). Like our study, large spatial variations were also found in previous studies. For example, Tang et al. found that GRSP decreased with increasing soil depth in farmland (1.60–2.94 mg·g^−1^), artificial grassland (1.82–3.18 mg·g^−1^), and orchard (1.41–1.91 mg·g^−1^) [7]. Decreasing GRSP with the increase of soil depth (0–40 cm) was found in the rhizosphere of a* Citrus unshiu* orchard, and the ranges of EE-GRSP and T-GRSP were, respectively, 0.3–0.6 mg·g^−1^ and 0.5–0.8 mg·g^−1^ [30]. Over 3-fold site variations together with land uses influences were also reported, and the order was secondary forest (3.47 mg·g^−1^) > paddy field (2.87 mg·g^−1^) > rubber plantation (2.27 mg·g^−1^) > orchard (1.73 mg·g^−1^ > sugarcane (1.03 mg·g^−1^) [31]. Even larger (16-fold) site variations in T-GRSP concentration were also found in different sites, with a good match with AMF activity [32]. About 1.5-fold spatial variations in GRSP concentration were also found between abandoned and active cultivation of olive groves [33]. GRSP concentration also differed in different soil size-fractions due to soil management [34]. All these studies manifested that land use changes, different sites, and vertical soil profiles induced large variations in GRSP concentration.

### 4.2. Compositional Clarifications of Purified GRSP and Its Spatial Variation: Replenishing Previous Studies

The composition of GRSP gets more and more attention in recent studies owing to its importance in exploring the function of GRSP in soil systems [9, 12]. Infrared spectroscopy, X-ray diffraction, and 3D fluorescence spectroscopy were used to characterize GRSP compositional traits in this paper, which is a complement of previous studies.

Infrared spectral scanning is suitable for the study of biological polymer structure and the polypeptide chain of configuration [23, 35, 36]. By using this technique, Schindler et al. found significant carboxylic functionality of GRSP [9], and we elucidated several functional traits in T-GRSP, that is, I: stretching of O–H, N–H, C–H (8050.9); II: C–H stretching (258.8); III: stretching of C=O, COO– (2039.9); IV: COO– stretching, C–H bending (500.9); V: C–O stretching and O–H bending (95.1); VI: stretching of C–O, Si–O–Si (1277.3); VII: O–H bending (25.7) (Figure 1, Table 4). This has laid the foundation for the future studies of GRSP, which is one of the new discoveries and complements in GRSP functional traits.

X-ray diffraction is suitable for the study of proteins of crystallization [12, 37]. Gillespie et al. used this technology to characterize the gross chemical structure of GRSP at the atomic and molecular scale [12]. This information is useful toward determining the kinds of materials released by the extraction protocol of GRSP [38, 39], thus providing assessments of the bulk composition of GRSP. Gillespie et al. also revealed that glomalin is a rich mixture of proteinaceous, humic, lipid, and inorganic substances [12]. By using this technique, diffraction peak position (2*θ* = 19.8°), average grain size (129.3 nm), and relative crystallinity (1.08%) of purified T-GRSP were firstly defined (Table 5) and these data will provide a basis for future studies of GRSP.

3D fluorescence spectroscopy was an effective method to study protein conformation in solution [40] and also detected dissolved organic matter fluorescence peaks [41]. Fluorescence detection in arbuscular mycorrhizal fungal structures and GRSP provided evidence of possible accumulation of Al in AM fungal structures and GRSP [42]. Fluorescent antibody was also used to detect hyphae and GRSP from AMF [43]. The 3D fluorescence spectra of GRSP in this paper proved that GRSP is a mixture of seven kinds of fluorescent substances: tyrosine-like protein, tryptophan-like protein, fulvic acid-like, soluble microbial byproduct-like, humic acid-like, nitrobenzoxadiazole-like, calcofluor white-like (Figure 2, Table 5). This finding replenished the discoveries of GRSP as a mixture of variable proteins and other substances [9].

Besides the concentration variations, compositional variations would be highly useful in identifying any ecological functions of GRSP in soils [9, 12], and we also quantified the site variations of these compositional traits in this paper (Tables 4 and 5). This study clearly manifested that significant spatial variations of GRSP were not only in its concentration, but also its compositional traits.

### 4.3. GRSP Concentration and Compositional Variations Were Regulated by Soil pH, While GRSP Concentration Was Related to Soil Bulk Density: Reason and Function from Regression Analysis

Although some studies tried to relate the concentration differences of T-GRSP and EE-GRSP to abiotic and biotic factors [8, 44], few papers have been devoted to the relations between GRSP compositional variations and soil properties. One of our important findings is that soil pH is mainly responsible for the observed differences both in GRSP concentration and compositional traits, while the regulations on soil physical properties (soil bulk density) are mainly from the GRSP amount (Table 2, Figures 3 and 4).

Previous study reported that the neutral or slightly acidic soil was suitable to the growth of plant roots and fungi [45]. Recent studies reported that soil pH negatively correlated with GRSP concentration [46–48]; our result also agreed with them. Moreover, correlations between soil pH and different infrared functional traits (aliphatic C–H stretching, positive correlation; C–O stretching and O–H bending, positive correlation; stretching of C–O, Si–O–Si, negative correlation) and relative crystallinity (negative correlation) were also generally observed. Soil pH directly affects the AM fungal formation [7], effectiveness of AMF for improving plant viability [49, 50], and synthesis and secretion of GRSP from AMF [51]. The finding of this paper manifested that the direct influences from pH on GRSP composition are on different functional groups from infrared spectrum as well as its X-ray features, such as crystallization. All these findings proved that soil acidity should be mainly responsible for variations in concentration and compositions of GRSP.

The role of GRSP in soil aggregate stability modification is well known [5]. Like previous study [49], the significant negative correlations between soil bulk density and GRSP concentration (T-GRSP, EE-GRSP) indicate their role in regulating soil structure. The relations between compositional traits (from infrared spectrum, X-ray diffraction, and 3D fluorescent spectrum) and soil bulk density were also tested in this paper, and none of them were statistical significant (figure not shown here). Thus, the concentration of GRSP, but not its compositional variations, mainly determines its function in soil structure modifications.

## 5. Conclusions

By using infrared spectrum, X-ray diffraction, and 3D fluorescent spectrum, we found the main infrared functional groups of GRSP, characteristic diffraction peak, relative crystallinity, grain size, and seven fluorescent substances. Like the large spatial variations in GRSP concentration, variations in compositional traits were quite large too. Soil pH changes were mainly responsible for these spatial variations. As a typical secretion from AMF, the GRSP-related findings are good for understanding the underlying mechanism of GRSP in soil functional maintenance.

## Figures and Tables

**Table 1:** Basic information of 6 sampling sites.

Location	Soil type	China soil taxonomy	Longitude	Latitude	Average altitude
Mingshui	Phaeozem	Pachic argi-udic Isohumosols	125°41′~126°42′	45°08′~45°43′	271.4 m
Lanling	Chernozem	Typic-calci-ustic argosols	125°13′~126°18′	45°13′~45°18′	400.0 m
Fuyu	Chernozem	Typic-calci-ustic argosols	124°48′~126°51′	45°37′~45°40′	162.4 m
Zhaozhou	Solonetz	Typic takyri-alkalic halosols	124°55′~125°12′	45°41′~45°49′	150.0 m
Zhaodong	Solonetz	Typic takyri-alkalic halosols	125°22′~126°22′	45°10′~46°20′	160.5 m
Dumeng	Cambosols	Typic dark aquic cambosols	124°19′~125°12′	45°46′~46°55′	146.0 m

**Table 2:** Two-way ANOVA results on spatial (site, soil layer) variations of different soil physicochemical properties in poplar shelterbelt forests in Songnen Plain.

Index	pH value	EC	Bulk density	Soil moisture
Site				
*F*	55.21	2.08	29.76	111.05
*P* value	0.000	0.068	0.000	0.000
Soil layer				
*F*	7.13	3.90	7.62	9.34
*P* value	0.000	0.004	0.000	0.000
Interaction				
*F*	0.26	0.56	1.55	1.22
*P* value	1.000	0.937	0.062	0.232

Site-related differences (mean and their statistical significance)				
Dumeng	8.49^a^	105.7^ab^	1.51^a^	5^d^
Fuyu	8.46^a^	152.1^a^	1.40^bc^	17^a^
Lanling	7.68^b^	122.6^ab^	1.43^b^	9^c^
Mingshui	7.44^b^	80.9^b^	1.32^d^	16^a^
Zhaodong	8.51^a^	114.9^ab^	1.39^bc^	13^b^
Zhaozhou	8.53^a^	139.5^ab^	1.38^c^	9^c^
Largest/lowest ratio	1.15	1.88	1.14	1.89

Soil layer-related differences (mean and their statistical significance)				
0–20 cm	8.08^ab^	105.2^b^	1.37^c^	13^a^
20–40 cm	8.00^b^	173.7^a^	1.39^bc^	12^ab^
40–60 cm	8.15^ab^	114.4^ab^	1.41^abc^	11^ab^
60–80 cm	8.32^a^	110.2^b^	1.43^ab^	10^b^
80–100 cm	8.38^a^	92.9^b^	1.43^a^	10^b^
Largest/lowest ratio	1.05	1.87	1.04	1.30

Mean of pooled data	8.19	119.28	1.41	11

Note: Different lowercases indicate the related differences between different soil layers or between different sites were statistically significant (*P* < 0.05).

**Table 3:** Two-way ANOVA results on spatial (site, soil layer) varaitions of GRSP (T-GRSP, EE-GRSP) concentration in poplar shelterbelt forests in Songnen Plain.

Index	T-GRSP concentration	T-GRSP storage	EE-GRSP concentration	EE-GRSP storage
Site				
*F*	62.75	54.94	65.59	57.53
*P* value	0.000	0.000	0.000	0.000
Soil layer				
*F*	64.87	59.21	39.32	35.32
*P* value	0.000	0.000	0.000	0.000
Interaction				
*F*	2.14	2.07	1.14	1.28
*P* value	0.003	0.005	0.310	0.187

Site-related differences (mean and their statistical significance)				
Dumeng	2.93^cd^	87.46^cd^	0.48^b^	14.32^b^
Fuyu	2.65^d^	71.66^d^	0.38^bc^	10.40^bc^
Lanling	4.05^c^	115.00^c^	0.30^cd^	8.78^cd^
Mingshui	6.60^a^	172.57^a^	0.87^a^	22.88^a^
Zhaodong	5.25^b^	143.91^b^	0.22^d^	6.01^d^
Zhaozhou	2.10^d^	56.76^d^	0.33^cd^	8.94^cd^
Largest/lowest ratio	3.14	3.04	3.95	3.81

Soil layer-related differences (mean and their statistical significance)				
0–20 cm	6.29^a^	169.83^a^	0.67^a^	18.26^a^
20–40 cm	4.51^b^	123.07^b^	0.51^b^	14.06^b^
40–60 cm	3.81^bc^	105.25^bc^	0.38^bc^	10.50^bc^
60–80 cm	2.92^cd^	81.82^cd^	0.33^c^	9.20^c^
80–100 cm	2.11^d^	59.50^d^	0.26^c^	7.42^c^
Largest/lowest ratio	2.98	2.85	2.58	2.46

Mean of pooled data	3.93	107.89	0.43	11.89

Note: Different lowercases indicate the related differences between different soil layers or between different sites were statistically significant (*P* < 0.05).

**Table 4:** Differences of absorption peak area of functional traits of T-GRSP at different sites in Songnen Plain.

Site	The absorption peak area of functional traits of different wave numbers						
	3750−3000 cm^−1^	3000−2820 cm^−1^	1750−1480 cm^−1^	1480−1320 cm^−1^	1320−1200 cm^−1^	1200−930 cm^−1^	930−840 cm^−1^
Dumeng	7293.0 (634.2)	331.3 (115.8)	1949.7 (185.1)	520.7 (92.9)	102.3 (34.5)	1150.0 (154.1)	26.7 (9.6)
Fuyu	8052.3 (727.2)	291.3 (99.0)	2019.3 (56.9)	444.0 (29.8)	113.0 (42.5)	1187.7 (47.9)	17.3 (1.2)
Lanling	8559.0 (1235.2)	176.3 (24.1)	2045.7 (354.9)	483.3 (67.7)	59.0 (12.5)	1437.0 (240.9)	24.0 (7.5)
Mingshui	8489.3 (379.2)	185.3 (10.1)	2083.0 (137.5)	536.7 (89.8)	65.7 (21.1)	1508.3 (103.8)	31.3 (11.2)
Zhaodong	8203.7 (939.4)	301.3 (85.5)	2181.7 (165.3)	567.3 (58.5)	114.0 (25.5)	1205.3 (135.7)	18.3 (1.5)
Zhaozhou	7708.0 (623.5)	267.3 (46.3)	1960.0 (162.8)	453.3 (62.9)	116.7 (26.1)	1175.7 (134.4)	36.3 (11.0)

Largest/lowest ratio	1.17	1.88	1.12	1.28	1.98	1.31	2.10
Mean of pooled data	8050.9 (813.1)	258.8 (86.7)	2039.9 (185.2)	500.9 (74.7)	95.1 (34.3)	1277.3 (190.8)	25.7 (9.8)

**Table 5:** Differences of compositional parameters of purified T-GRSP of different sites in Songnen Plain. The data were derived from X-ray diffraction, ultraviolet spectrum, and 3D fluorescent spectrum.

Site	X-ray diffraction		Wavelength at 295 nm		Compounds derived from 3D fluorescence spectrum						
	Grain size (nm)	Relative crystallinity (%)	OD value from ultraviolet spectrum	Fluorescence intensity at the maximum emission wavelength	Tyrosine-like protein	Tryptophan-like protein	Fulvic acid-like	Soluble microbial byproduct-like	Humic acid-like	Nitrobenzoxadiazole-like	Calcofluor white-like
Lanling	125	1.35	1.14	28.28	6.41	6.39	13.14	—	33.78	51.88	52.51
Zhaodong	98	0.7	1.02	20.53	1.74	2.85	9.32	—	31.77	52.37	50.07
Dumeng	174	0.73	1.05	29.52	5.11	5.82	11.84	3.16	42.18	—	59.17
Zhaozhou	130	0.69	1.21	25.74	7.78	5.84	11.62	6.45	34.33	57.91	—
Fuyu	138	1.05	1.27	30.49	6.46	8.76	15.93	—	35.95	51.43	52.88
Mingshui	111	1.98	1.37	22.57	4.23	5.63	10.4	—	25.7	41.3	—

Largest/lowest ratio	1.41	2.71	1.34	1.49	4.5	3.07	1.71	2.04	1.64	1.4	1.18
Mean of pooled data	129 (2.6)	1.08 (0.5)	1.18 (0.13)	26.20 (3.98)	5.29 (2.1)	5.88 (1.9)	12 (2.31)	1.6 (2.69)	33.95 (5.4)	42.48 (21.5)	35.77 (27.9)
